# Ankylosis of the hips and knees due to sickle cell disease

**DOI:** 10.12688/f1000research.1-32.v1

**Published:** 2012-10-19

**Authors:** Saad Saleh Abdullah Al Elayan, Abdullah Al Hamdan

**Affiliations:** 1 Department of Orthopedics, Prince Sultan Military Medical City, Riyadh, 11612, Saudi Arabia

## Abstract

This is a case report of a 29-year-old Saudi male with sickle cell disease (SCD) with severe stiffness of his joints, mainly both knees and hips, secondary to complications of SCD. He was severely crippled: unable to sit, stand or walk, and was bedridden for 8 years when he was presented to us. Radiographs showed fusion of both knees and hips. There was no evidence of active osteomyelitis by Gallium scan. The patient’s hemoglobin S decreased to levels below 30% by exchange transfusion. Bilateral total hip replacement, as well as unilateral total knee replacement, was carried out to improve his level of function. There is only one reported case of such severe and multiple joint complications in a single patient suffering from SCD.

The increased life expectancy that medical advances have offered to the sickle-cell patients has led to the appearance of sickle-cell-related complications, which were previously only seen rarely. These complications were successfully managed and the patient was able to move and transfer using a wheel chair.

## Introduction

Sickle cell disease (SCD) is an autosomal recessive genetic disorder characterized primarily by chronic anemia and periodic episodes of pain, affecting millions throughout the world ^1^. SCD patients are at increased risk of bony complications of the disease, such as osteomyelitis, osteonecrosis, osteopenia and ankylosis ^2^. SCD is an important public health concern in the Kingdom of Saudi Arabia (KSA), particularly in the Eastern and South West provinces ^3^. Painful crises and avascular necrosis of the femoral head are common complications observed in these regions ^3^. This case presentation is that of a patient with severe bony complications resulting from his SCD. Total bilateral hip and unilateral knee arthroplasty were performed to correct hip and knee ankylosis secondary to SCD. To our knowledge, total bilateral hip and unilateral knee arthroplasty has not been described previously in the literature except in one case of an African male with a similar presentation ^4^.

## Case report

A 29-year-old Saudi male with SCD, was referred from the general surgery service to improve his poor physical condition. He was severely crippled and bed bound for 8 years with severe bilateral knee and hip dysfunction secondary to complications of SCD. Past history included an admission to hospital 8 years before for fever, swelling and severe pain of the right knee. Needle aspiration, as well as irrigation and debridement, was performed and the patient was diagnosed with septic arthritis. Since then he had developed progressive joint stiffness involving the dorsal and lumbar spines as well as the lower extremity.

On physical examination his knee range of motion (ROM) bilaterally was almost nonexistent with the knees held in full extension. His hips ROM was also severely limited, with no ROM. Loss of hip flexion was noted to result in the most severe functional loss with the hips fixed in full extension, adduction of 15° and 50° of external rotation. He required a 2-person assist as well as a walker to weight bear however he was unable to mobilize.

**Figure 1.**
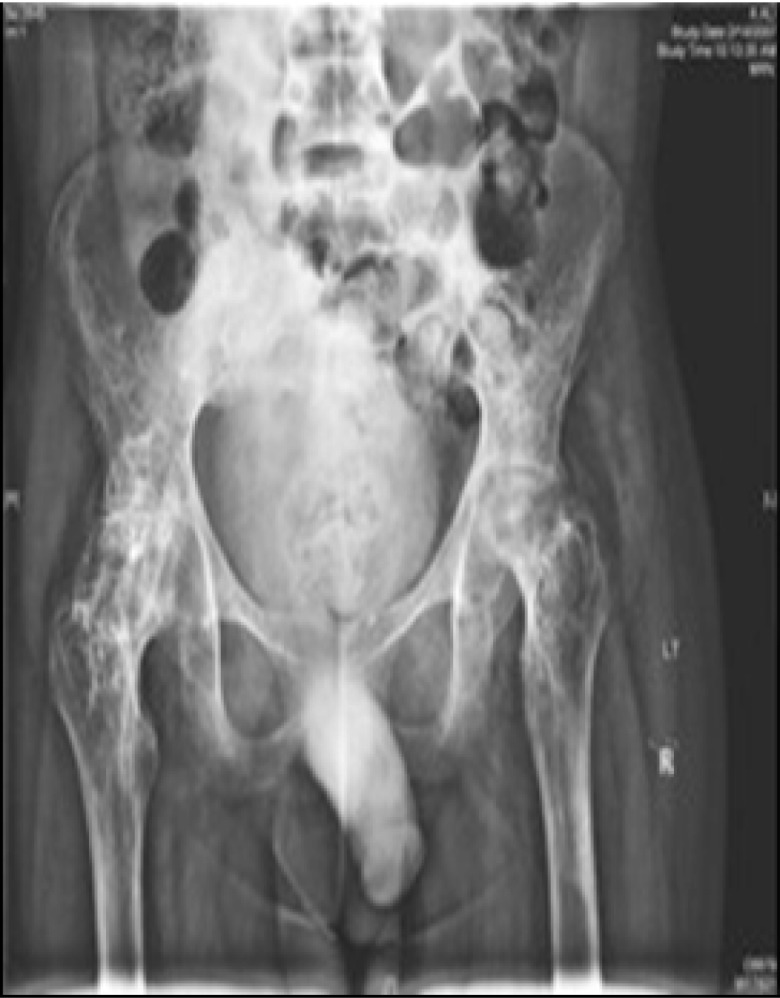
AP Hips x-rays showed severe avascular necrosis and ankylosis.

**Figure 2.**
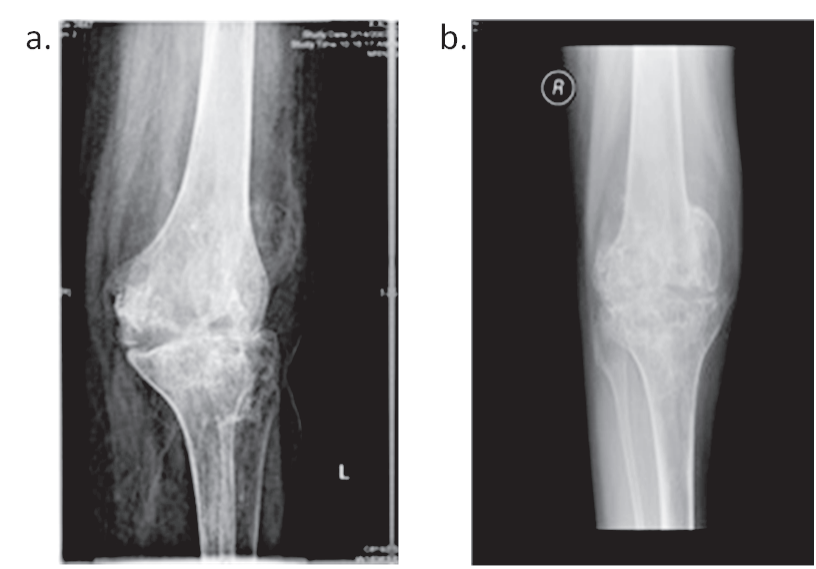
AP left & right knee x-ray shows erosion and ankylosis.

Serial radiological studies were done. Radiological investigations demonstrated severe avascular necrosis and ankylosis of the hip ( Figure 1) and severe erosion of the articular surfaces of the knees as well as ankylosis ( Figure 2). Computed tomography (CT) scan of the hips and knees showed similar findings as that of the X-rays. Shoulders and spine X-ray were done for further assessment. Gallium bone scan demonstrated no evidence of active osteomyelitis. Our patient had a proper preoperative evaluation and blood transfusion to prevent adverse outcomes and sickle cell complications postoperatively ^5^. We took him to surgery for bilateral cementless total hip replacement (THR) in one session.

**Figure 3.**
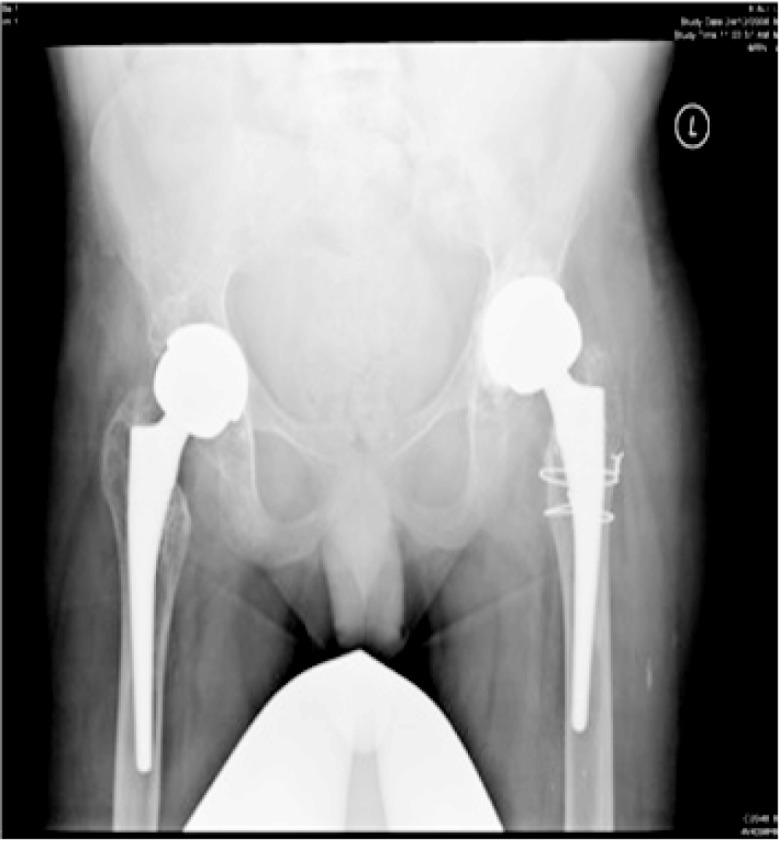
AP pelvis x-ray post bilateral THR.

The patient was operated on with an aim to provide movement at the hips and knees and to recover his ability to sit, stand, transfer, balance and improves personal hygiene. Bilateral THR was done successfully ( Figure 3). The procedures were performed utilizing the Harding lateral approach. Particular attention was paid to padding bony prominences and skin care in view of his poor skin condition. Intraoperatively, the bone was noted to be very fragile. The patient was kept in the intensive care unit for two days post operatively for observation and pain control. Post bilateral THR, the patient had marked improvement of hip flexion bilaterally permitting easy functional sitting. Post surgery physiotherapy resulted in great improvement in his general physical activity.

**Figure 4.**
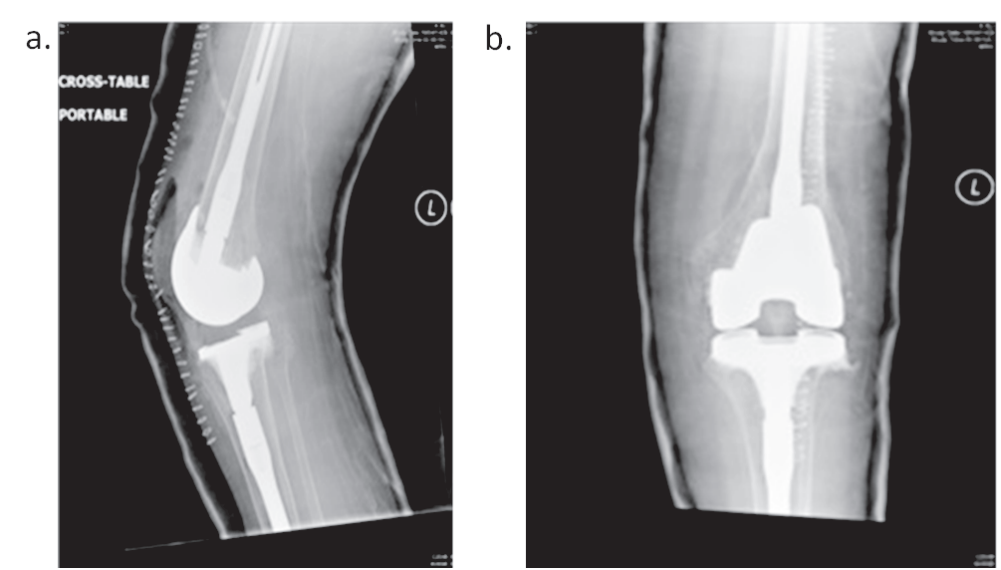
X-ray of left knee post TKR.

Left total knee replacement (TKR) was performed one month after the THR ( Figure 4). Intraoperatively, the bone quality was noted to be severely deficient. The quadriceps muscle was adherent to the femur and atrophied; therefore, V-Y quadricepsplasty was done. Soft tissue was mobilized carefully around the joint prior to component insertion. Intraoperatively, knee flexion approached 50°. Post-left-TKR rehabilitation was severely restricted secondary to poor bone quality. This prevented adequate ROM exercises and function, and ROM gains at the knee were minimal. Poor bone quality also limited the ability for the patient to progress to functional lower extremity weight-bearing activities. Postoperatively, rehabilitation, patient education, transfer training and functional rehabilitation were carried out. This allowed the patient to transfer safely and independently from bed to wheel chair without much pain, and improved the quality of life by changing the patient’s functional ability and allowing the freedom to mobilize independently with a wheelchair.

## Discussion

Unlike normal red blood cells (RBC), sickled cells, due to abnormal morphology, are unable to negotiate small blood vessels resulting in arterial occlusion and subsequent ischemia. This process can ultimately damage tissues and vital organs. The prevalence of avascular necrosis (AVN) in sickle-cell-disease patients is increasing, especially with the increased life expectancy in these patients. It is believed that the AVN results from repeated episodes of localized areas of epiphyseal, metaphyseal and diaphyseal bone marrow infarctions, resulting in ingrowths of new bone as well as diffuse sclerosis resulting from vascular occlusion by sludging of sickle cells in the sinusoids. In general, the bilateral simultaneous THR has demonstrated better functional outcome than the staged procedure in patients with the same condition, with no significant increase in the rate of dislocation or thromboembolic events ^6^. A similar case was published in 2000: a Congolese sickle cell patient was referred to Belgium for management of severe stiffness of all his major joints. That patient was managed by bilateral staged girdle stone procedure initially, with a subsequent surgical site infection of the left hip. The infection was managed with appropriate antibiotics and debridement. Once the infection was treated, the patient had a staged bilateral THR procedure. The patient regained his ability to walk with crutches after the surgeries ^4^. Another case was reported in 2008: a 25-year-old female with severe ankylosis of her hips and knees secondary to rheumatoid arthritis, and her function was severely limited. She underwent staged bilateral THR, followed by a staged bilateral TKR, the functional outcome was excellent in that case as well ^7^.

The increased life expectancy that medical advances have offered to the sickle-cell patients has led to the appearance of sickle-cell-related complications, which were previously only seen rarely. Orthopedic surgeons should be aware of the optimal management options as well as possible operative and postoperative complications of patients with this disease.

## Consent

Written consent was obtained from the patient and the next of kin for the publication of the clinical details and clinical images related to the case report.
