# Supplementary material for: Ankylosis of the hips and knees due to sickle cell disease
Source: F1000Res. 2012 Oct 19;1:32. [Version 1] doi: 10.12688/f1000research.1-32.v1 (PMC3814913; doi:10.12688/f1000research.1-32.v1)
Supplement: Peer review history

# Peer review: Referee response for version 1

**Published:** 2012-11-29

**Authors:** Adlette Inati

**Affiliations:** 1 Division of Pediatric Hematology and Oncology, Children's Center for Cancer and Blood Diseases, Rafik Hariri University Hospital, Beirut, Lebanon

Avascular bone necrosis is a debilitating complication of sickle cell disease (SCD). Due to the prolonged life expectancy of SCD patients, the incidence of this complication is surging as it could be a manifestation of the disease chronicity.

In this case report, The authors present a severe case of ankylosis of hips and knees secondary to SCD. Indeed, the severity of this presentation is unusual and may be partially due to lack of prevention and optimal early management. Although the clinical management of the patient was not discussed, it would be interesting to know if this patient was on hydroxyurea or on antithrombotic agents and if he was enrolled in a comprehensive SCD care facility. Moreover, since the patient has been bedridden for eight years, it is possible that thrombophilic factors may have further exacerbated the condition and its progression to multiple joints.

I have read this submission. I believe that I have an appropriate level of expertise to confirm that it is of an acceptable scientific standard.

# Peer review: Referee response for version 1

**Published:** 2012-10-25

**Authors:** Maria Luisa Brandi

**Affiliations:** 1 Department of Internal Medicine, University of Florence, Viale Morgagni, Florence, Italy

This case report represents some very nice work.

I have read this submission. I believe that I have an appropriate level of expertise to confirm that it is of an acceptable scientific standard.
